# Correlation between nerve conduction velocity abnormality patterns and clinical severity grading in chemotherapy-induced peripheral neuropathy: a retrospective cohort study

**DOI:** 10.3389/fneur.2026.1778979

**Published:** 2026-06-04

**Authors:** Dandan Xu, Xiaofei Lan, Luhan Chen, Li Zhang

**Affiliations:** 1 Department of Neurology, Jiaxing Second Hospital, Jiaxing, China; 2 Department of Inspection, Jiaxing Institute of Food, Drug and Product Quality Inspection and Testing, Jaixing, China; 3 Department of Operating Room, Jiaxing Second Hospital, Jiaxing, China

**Keywords:** biomarker, chemotherapy-induced peripheral neuropathy, clinical severity, electrophysiology, nerve conduction study

## Abstract

**Objective:**

Chemotherapy-induced peripheral neuropathy (CIPN) is a common and debilitating adverse effect of neurotoxic chemotherapy, yet the longitudinal relationship between nerve conduction study (NCS) changes and clinical severity remains insufficiently defined.

**Methods:**

In this retrospective cohort of 186 patients undergoing serial clinical and electrophysiological assessment at baseline, mid-treatment, end-of-treatment, and follow-up, sural sensory nerve action potential (SNAP) amplitude showed the strongest inverse association with end-of-treatment NCI-CTCAE severity (*r* = −0.724, *P* < 0.001).

**Results:**

Absolute end-of-treatment sural SNAP amplitude identified Grade ≥2 CIPN with an area under the curve (AUC) of 0.856, while a relative decline from baseline of ≥35% yielded an AUC of 0.872. Patients requiring neurotoxicity-related dose modification had larger early sural SNAP reductions from baseline to mid-treatment than those without treatment change (34.8% ± 13.2% vs. 17.6% ± 11.4%, *P* < 0.001). Four electrophysiological phenotypes were observed and were associated with differential clinical severity and recovery trajectories.

**Conclusions:**

These findings support serial large-fiber NCS, particularly sural SNAP amplitude and its percentage change from baseline, as objective correlates of CIPN severity and treatment tolerance in routine clinical practice.

## 1 Introduction

Chemotherapy-induced peripheral neuropathy (CIPN) represents one of the most prevalent and debilitating complications of cancer treatment, affecting 30% to 70% of patients receiving neurotoxic chemotherapy agents (1). The condition significantly impacts cancer survivors' quality of life, often persisting for months or years after treatment completion, and may necessitate dose modifications or premature discontinuation of effective chemotherapy regimens (2). With the growing population of cancer survivors—estimated at over 18 million in the United States alone as of 2022—the long-term burden of CIPN has become an increasingly important public health concern (3).

The pathophysiology of CIPN involves complex mechanisms that vary according to the specific chemotherapeutic agent. Taxanes, including paclitaxel and docetaxel, induce peripheral neuropathy primarily through microtubule stabilization, disrupting axonal transport and causing dorsal root ganglion (DRG) neuronal injury (4). This results in a length-dependent axonal sensory neuropathy characterized by the “dying back” pattern of axonal degeneration (5). Platinum-based compounds, such as oxaliplatin and cisplatin, preferentially accumulate in DRG neurons, causing DNA damage and mitochondrial dysfunction that leads to sensory neuronopathy (6). The clinical manifestations include numbness, tingling, burning pain, and sensory loss in a characteristic stocking-glove distribution, with motor involvement occurring in more severe cases (7).

Despite its clinical significance, standardized assessment of CIPN severity remains challenging. The National Cancer Institute Common Terminology Criteria for Adverse Events (NCI-CTCAE) is the most widely used grading system in clinical practice and research (8). However, this clinician-rated scale has demonstrated limitations including low inter-rater reliability and reduced sensitivity to subtle changes in neuropathy severity (9). The total neuropathy score (TNS) and its clinical version (TNSc) offer more comprehensive neurological assessments but require specialized training and additional time for administration (10). Patient-reported outcome measures (PROMs), such as the European Organization for Research and Treatment of Cancer Quality of Life Questionnaire-CIPN20 (EORTC QLQ-CIPN20), capture subjective symptom experiences but may not correlate consistently with objective neurological findings (11).

Nerve conduction studies (NCS) provide objective, quantitative assessments of peripheral nerve function and represent the gold standard for diagnosing peripheral neuropathies (12). In CIPN, characteristic electrophysiological findings include reduced sensory nerve action potential (SNAP) amplitudes with relative preservation of conduction velocities, consistent with axonal pathology (13). Previous studies have demonstrated that SNAP amplitude decreases often precede clinical symptom onset in patients receiving neurotoxic chemotherapy (14). However, the relationship between specific NCS abnormality patterns and clinical severity grading remains incompletely characterized, and the utility of electrophysiological parameters as severity biomarkers requires further validation.

The correlation between objective electrophysiological measures and clinical severity assessments has important implications for longitudinal phenotyping and risk stratification in CIPN. Because the present study was retrospective and NCS results were not used to direct treatment in real time, the aim was to evaluate clinically relevant associations between serial electrophysiological changes, symptom severity, and treatment tolerance rather than to test a prospective intervention strategy (12–15).

This retrospective cohort study aimed to investigate the association between NCS abnormality patterns and clinical severity grading in patients with CIPN, to examine whether baseline and early longitudinal NCS changes were associated with subsequent severe neuropathy and neurotoxicity-related dose modification, and to characterize distinct electrophysiological phenotypes and their recovery profiles.

## 2 Materials and methods

### 2.1 Study design and patient population

This retrospective cohort study was conducted at the Department of Neurology and the Cancer Center of a tertiary academic medical center. Medical records and electrophysiological databases were reviewed for patients who received neurotoxic chemotherapy between January 2021 and December 2024. The Jiaxing Second Hospital review board approved this retrospective analysis and waived additional study-specific written informed consent because only de-identified routinely collected clinical data were analyzed. In our center, NCS referral was not performed as universal screening; patients were referred when they developed new sensory complaints, neuropathic pain, gait imbalance, hand clumsiness, or when the treating oncologist or neurologist considered objective documentation necessary before continuing potentially neurotoxic chemotherapy. The cohort therefore represents a clinically referred population rather than an unselected chemotherapy population.

Eligible patients were adults aged 18 years or older with histologically confirmed malignancies who had received neurotoxic chemotherapy regimens containing taxanes (paclitaxel, docetaxel, or nab-paclitaxel) or platinum-based agents (oxaliplatin, cisplatin, or carboplatin). Patients were required to have had an Eastern Cooperative Oncology Group (ECOG) performance status of 0–2 at treatment initiation and adequate organ function to receive planned chemotherapy. Exclusion criteria included: (1) pre-existing peripheral neuropathy of any etiology documented in medical records, including diabetic neuropathy, hereditary neuropathy, or prior chemotherapy-induced neuropathy; (2) baseline NCI-CTCAE peripheral sensory neuropathy grade ≥1; (3) concurrent or prior treatment with other known neurotoxic agents within 6 months; (4) medical conditions potentially causing neuropathy, including uncontrolled diabetes mellitus (hemoglobin A1c > 8%), vitamin B12 deficiency (< 200 pg/ml), thyroid dysfunction, chronic kidney disease (estimated glomerular filtration rate < 30 ml/min/1.73 m^2^), or chronic alcohol use disorder; (5) incomplete neurological assessments or nerve conduction studies; and (6) documented life expectancy < 6 months at treatment initiation.

Sample size calculation was based on the primary objective of detecting a correlation coefficient of at least 0.3 between sural SNAP amplitude and NCI-CTCAE grade, with 90% power and a two-sided significance level of 0.05. A minimum of 180 patients with complete data was required for analysis.

### 2.2 Chemotherapy regimens and treatment protocols

Patients had received chemotherapy according to standard institutional protocols and national guidelines. Taxane-based regimens included: (1) paclitaxel 175 mg/m^2^ intravenously every 3 weeks or 80 mg/m^2^ weekly; (2) docetaxel 75–100 mg/m^2^ every 3 weeks; and (3) nab-paclitaxel 260 mg/m^2^ every 3 weeks or 100–125 mg/m^2^ weekly. Platinum-based regimens included: (1) oxaliplatin 85–130 mg/m^2^ every 2 or 3 weeks; (2) cisplatin 60–75 mg/m^2^ every 3 weeks; and (3) carboplatin dosed at an area under the concentration-time curve of 5–6 mg/ml.min every 3 weeks. Combination regimens comprised concurrent taxane and platinum administration. Cumulative dose intensity was recorded for each patient.

Dose modifications for neurotoxicity had been performed according to established guidelines. For Grade 2 sensory neuropathy, chemotherapy was continued with close monitoring and consideration for dose reduction (25% reduction for subsequent cycles). For Grade 3 sensory neuropathy or Grade 2 motor neuropathy, treatment was held until recovery to Grade ≤ 1, then resumed with dose reduction. Grade 4 neuropathy or failure to recover to Grade ≤ 1 within 4 weeks resulted in treatment discontinuation.

### 2.3 Clinical severity assessment

Clinical assessment data were extracted from medical records for baseline (within 7 days before chemotherapy initiation), mid-treatment (after completion of approximately 50% of planned cycles), end-of-treatment (within 14 days of the last chemotherapy dose), and 3-month and 6-month follow-up visits when available. At our institution, NCS was scheduled at the same longitudinal time points whenever a patient had an electrophysiological evaluation, and paired clinical-NCS analyses in the present study were restricted to visits with both assessments completed within the same 7-day window.

The NCI-CTCAE version 5.0 peripheral sensory neuropathy and peripheral motor neuropathy scales were used as the primary clinical severity measures. Sensory neuropathy was graded as follows: Grade 1, asymptomatic or mild symptoms not interfering with function; Grade 2, moderate symptoms limiting instrumental activities of daily living (ADLs); Grade 3, severe symptoms limiting self-care ADLs; and Grade 4, life-threatening consequences requiring urgent intervention. Motor neuropathy grading followed analogous criteria based on weakness severity and functional impact.

The Total Neuropathy Score-clinical version (TNSc) data were extracted when available. The TNSc comprises seven items: (1) sensory symptoms, (2) motor symptoms, (3) autonomic symptoms, (4) pin sensibility, (5) vibration sensibility, (6) strength, and (7) deep tendon reflexes. Each item is scored from 0 (normal) to 4 (severely abnormal), yielding a total score ranging from 0 to 28, with higher scores indicating more severe neuropathy. The TNSc was categorized into severity grades: 0–4 (mild), 5–10 (moderate), 11–16 (moderately severe), and 17–28 (severe).

Patient-reported outcome data using the EORTC QLQ-CIPN20 questionnaire were extracted when available. This questionnaire comprises 20 items evaluating sensory symptoms (9 items), motor symptoms (8 items), and autonomic symptoms (3 items). Responses are scored on a 4-point Likert scale (1 = “not at all” to 4 = “very much”), with scores linearly transformed to a 0–100 scale. Higher scores indicate more severe symptoms.

### 2.4 Nerve conduction studies

Comprehensive longitudinal NCS data were extracted from the electrophysiological database. All studies had been performed by certified electrophysiology technicians under the supervision of board-certified neurophysiologists, using standardized protocols with a Nihon Kohden MEB-2300 electromyography system with standardized filter settings (sensory: 20–2,000 Hz; motor: 2–10,000 Hz). Limb temperature had been maintained at >=32 °C using warming devices when necessary, and skin temperature was documented for each recording. Baseline, mid-treatment, end-of-treatment, 3-month, and 6-month examinations were analyzed when available.

Sensory NCS had been performed on the following nerves bilaterally: (1) sural nerve-antidromic technique with recording electrodes placed posterior and inferior to the lateral malleolus and stimulation 14 cm proximally along the posterior calf; (2) median nerve-antidromic technique with ring electrodes on the index finger and wrist stimulation at a fixed 14 cm distance; and (3) ulnar nerve-antidromic technique with ring electrodes on the fifth finger and wrist stimulation at a fixed 14 cm distance. The following parameters were extracted: SNAP amplitude (baseline-to-peak, uV), SNCV (m/s), and peak latency (ms).

Motor NCS had been performed on: (1) peroneal nerve-recording from extensor digitorum brevis with stimulation at the ankle, below the fibular head, and above the fibular head; (2) tibial nerve-recording from abductor hallucis with stimulation at the ankle and popliteal fossa; and (3) median nerve-recording from abductor pollicis brevis with stimulation at the wrist and elbow. Parameters extracted included: CMAP amplitude (baseline-to-peak, mV), MNCV (m/s), distal motor latency (ms), and minimal F-wave latency (ms).

Reference values were age- and height-adjusted according to laboratory-specific normative data. Abnormalities were defined as values exceeding 2 standard deviations from the mean or falling below the 5th percentile of normative values. For amplitude parameters, a reduction of ≥50% from baseline was considered significant axonal loss. A composite NCS abnormality score was calculated by summing the number of abnormal parameters across all tested nerves (range 0–24).

### 2.5 Electrophysiological phenotype classification

Based on the pattern of NCS abnormalities, patients with CIPN were classified into four electrophysiological phenotypes: (1) Pure sensory axonopathy—reduced SNAP amplitudes with preserved SNCV ( ≤ 10% reduction) and normal motor studies; (2) Mixed sensorimotor axonopathy—reduced SNAP and CMAP amplitudes with preserved conduction velocities; (3) Sensory neuronopathy pattern—non-length-dependent SNAP amplitude reduction affecting upper and lower extremities equally or more severely in upper extremities, with abnormal median/ulnar SNAP preceding sural abnormalities; and (4) Demyelinating features—SNCV or MNCV reduction >20% from baseline or below normal limits, with relatively preserved amplitudes, or evidence of conduction block or temporal dispersion.

### 2.6 Statistical analysis

Statistical analyses were performed using SPSS version 27.0 (IBM Corporation, Armonk, NY, USA) and R version 4.2.0. Continuous variables were expressed as mean +/– standard deviation (SD) for normally distributed data or median with interquartile range (IQR) for non-normally distributed data. Categorical variables were presented as frequencies and percentages. Normality was assessed using the Shapiro-Wilk test. Longitudinal analyses used paired data from the same participant at predefined time points; the number of available paired observations was reported for each analysis.

Comparisons between groups were performed using one-way analysis of variance (ANOVA) with *post-hoc* Tukey's test for normally distributed continuous variables, Kruskal–Wallis test with Dunn's *post-hoc* test for non-normally distributed variables, and chi-square test or Fisher's exact test for categorical variables. Correlations between NCS parameters and clinical severity measures were evaluated using Pearson's correlation coefficient for normally distributed variables and Spearman's rank correlation coefficient for ordinal or non-normally distributed variables.

Receiver operating characteristic (ROC) curve analysis was performed to evaluate the discriminative ability of NCS parameters for identifying severe CIPN (Grade >=2). The area under the curve (AUC), optimal cutoff values (determined by Youden's index), sensitivity, specificity, positive predictive value (PPV), and negative predictive value (NPV) were calculated. In addition to absolute end-of-treatment values, we analyzed percentage change from baseline to mid-treatment and from baseline to end-of-treatment for sural SNAP amplitude. DeLong's test was used to compare AUCs between different parameters.

Multivariate logistic regression analysis was performed to identify independent factors associated with severe CIPN, with adjustment for potential confounders including age, sex, body mass index (BMI), controlled diabetes mellitus without clinical diabetic neuropathy, chemotherapy type, and cumulative dose. Diabetes status was defined as a documented history of diabetes with HbA1c < 8.0% and no neuropathic symptoms, signs, or prior electrophysiological evidence of diabetic neuropathy at baseline. Odds ratios (ORs) with 95% confidence intervals (CIs) were reported. Model performance was assessed using the Hosmer–Lemeshow goodness-of-fit test, Nagelkerke R2, and calibration plots.

Time-to-event analyses for CIPN development were performed using Kaplan-Meier curves with log-rank tests for comparison. Cox proportional hazards regression was used to identify factors associated with time to severe CIPN development. A two-tailed *P*-value < 0.05 was considered statistically significant.

## 3 Results

### 3.1 Patient characteristics

A total of 218 patients' records were reviewed for eligibility, of whom 186 met inclusion criteria and were included in the analysis. The baseline demographic and clinical characteristics are presented in Table 1. The mean age was 58.4 ± 11.2 years, and 124 (66.7%) patients were female. The most common primary malignancies were breast cancer (*n* = 68, 36.6%), colorectal cancer (*n* = 52, 28.0%), ovarian cancer (*n* = 32, 17.2%), and lung cancer (*n* = 22, 11.8%). Chemotherapy regimens included taxane-based therapy in 86 patients (46.2%), platinum-based therapy in 62 patients (33.3%), and combination taxane-platinum therapy in 38 patients (20.4%).

**Table 1:** Baseline demographic and clinical characteristics of study participants.

Characteristic	Total (*N* = 186)	CIPN developed (*n* = 142)	No CIPN (*n* = 44)	*P*-value
Age, years, mean ± SD	58.4 ± 11.2	59.8 ± 10.8	53.9 ± 11.5	0.002
Female, *n* (%)	124 (66.7)	98 (69.0)	26 (59.1)	0.224
BMI, kg/m^2^, mean ± SD	24.6 ± 4.2	25.1 ± 4.4	23.2 ± 3.3	0.009
Diabetes mellitus, *n* (%)	28 (15.1)	24 (16.9)	4 (9.1)	0.206
Hypertension, *n* (%)	62 (33.3)	51 (35.9)	11 (25.0)	0.180
Primary malignancy, *n* (%)				
Breast cancer	68 (36.6)	54 (38.0)	14 (31.8)	0.156
Colorectal cancer	52 (28.0)	42 (29.6)	10 (22.7)	
Ovarian cancer	32 (17.2)	24 (16.9)	8 (18.2)	
Lung cancer	22 (11.8)	14 (9.9)	8 (18.2)	
Other	12 (6.5)	8 (5.6)	4 (9.1)	
Chemotherapy regimen, *n* (%)				
Taxane-based	86 (46.2)	62 (43.7)	24 (54.5)	0.018
Platinum-based	62 (33.3)	46 (32.4)	16 (36.4)	
Taxane + Platinum	38 (20.4)	34 (23.9)	4 (9.1)	
ECOG performance status 0–1, *n* (%)	168 (90.3)	126 (88.7)	42 (95.5)	0.182
Baseline sural SNAP, μV, mean ± SD	12.4 ± 4.8	11.8 ± 4.6	14.2 ± 5.1	0.004
Baseline sural SNCV, m/s, mean ± SD	48.6 ± 5.2	47.9 ± 5.4	50.8 ± 4.2	0.001

BMI, body mass index; CIPN, chemotherapy-induced peripheral neuropathy; ECOG, Eastern Cooperative Oncology Group; SD, standard deviation; SNAP, sensory nerve action potential; SNCV, sensory nerve conduction velocity.

### 3.2 CIPN incidence and clinical severity distribution

**Figure 1:**
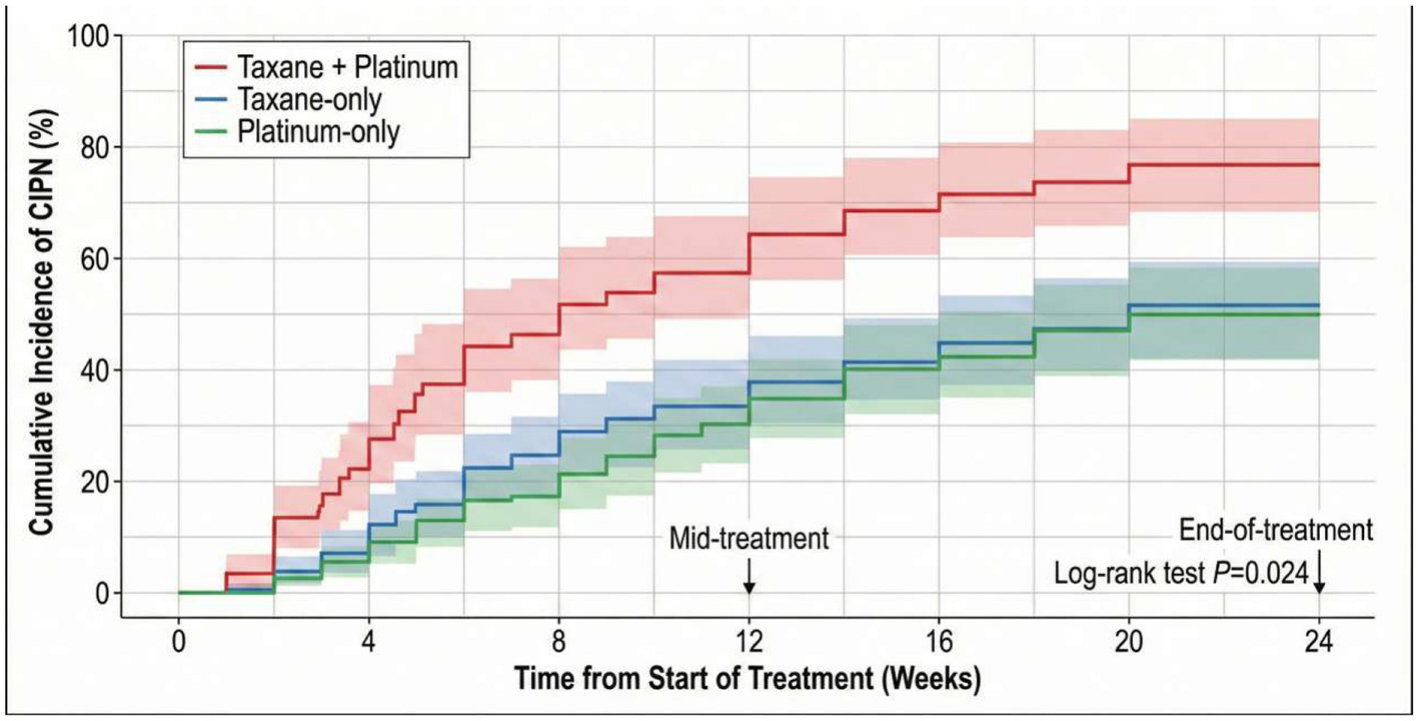
Cumulative incidence of CIPN according to treatment phase and chemotherapy regimen.

During the study period, 142 of 186 patients (76.3%) developed CIPN of any grade. The cumulative incidence increased progressively with treatment cycles, reaching 42.5% at mid-treatment and 76.3% at end-of-treatment (Figure 1). Neurotoxicity-related chemotherapy dose modification occurred in 38 patients (20.4% of the full cohort; 26.8% of patients with CIPN), including 29 dose reductions, 6 treatment delays, and 3 permanent discontinuations. Dose modification was uncommon in Grade 0–1 CIPN (6/102, 5.9%) but frequent in Grade 2–3 CIPN (32/84, 38.1%; *P* < 0.001).

The median time to CIPN onset was 8.2 weeks (IQR: 5.4–12.6 weeks) overall, with significant differences between regimens: 6.8 weeks for combination therapy, 8.4 weeks for taxane-only, and 10.2 weeks for platinum-only regimens (*P* = 0.008). Longitudinal paired NCS data were available in 186 patients at baseline, 171 at mid-treatment, 186 at end-of-treatment, 128 at 3 months, and 116 at 6 months. TNSc scores at end-of-treatment correlated strongly with NCS abnormalities, particularly sural SNAP amplitude and composite NCS score.

### 3.3 Nerve conduction study findings according to clinical severity

Table 2 presents the end-of-treatment NCS parameters stratified by end-of-treatment NCI-CTCAE sensory neuropathy grade. Progressive deterioration of sensory nerve parameters was observed with increasing clinical severity. Sural SNAP amplitude showed the most pronounced reduction across severity grades, decreasing from 14.2 +/– 5.1 uV in Grade 0 to 2.38 +/– 1.45 uV in Grade 3 patients (83.2% relative reduction).

**Table 2:** Nerve conduction study parameters according to NCI-CTCAE sensory neuropathy grade.

Parameter	Grade 0 (*n* = 44)	Grade 1 (*n* = 58)	Grade 2 (*n* = 54)	Grade 3 (*n* = 30)	*P*-value
Sensory nerves					
Sural SNAP amplitude, μV	14.2 ± 5.1	8.52 ± 3.24	5.18 ± 2.56	2.38 ± 1.45	< 0.001
Sural SNCV, m/s	50.8 ± 4.2	47.6 ± 4.8	45.2 ± 5.4	42.8 ± 6.2	< 0.001
Median SNAP amplitude, μV	22.4 ± 8.6	18.2 ± 7.4	14.6 ± 6.2	10.8 ± 5.4	< 0.001
Median SNCV, m/s	54.2 ± 4.8	52.4 ± 5.2	50.6 ± 5.8	48.2 ± 6.4	< 0.001
Ulnar SNAP amplitude, μV	18.6 ± 7.2	15.4 ± 6.4	12.2 ± 5.6	8.6 ± 4.8	< 0.001
Ulnar SNCV, m/s	52.8 ± 4.6	51.2 ± 5.0	49.4 ± 5.4	46.8 ± 6.0	< 0.001
Motor nerves					
Peroneal CMAP amplitude, mV	4.8 ± 1.6	4.2 ± 1.4	3.6 ± 1.2	2.8 ± 1.0	< 0.001
Peroneal MNCV, m/s	48.2 ± 4.4	46.8 ± 4.6	45.4 ± 5.0	43.2 ± 5.6	< 0.001
Tibial CMAP amplitude, mV	8.4 ± 2.8	7.6 ± 2.4	6.4 ± 2.2	5.2 ± 1.8	< 0.001
Tibial MNCV, m/s	46.4 ± 4.2	45.2 ± 4.4	44.0 ± 4.8	42.4 ± 5.2	0.002
Composite NCS score	0.8 ± 1.2	4.6 ± 2.4	8.4 ± 3.2	14.2 ± 4.6	< 0.001

CMAP, compound muscle action potential; MNCV, motor nerve conduction velocity; NCI-CTCAE, National Cancer Institute Common terminology criteria for adverse events; NCS, nerve conduction studies; SNAP, sensory nerve action potential; SNCV, sensory nerve conduction velocity.

Conduction velocities showed more modest reductions, with sural SNCV decreasing from 50.8 +/– 4.2 m/s in Grade 0 to 42.8 +/– 6.2 m/s in Grade 3 patients (15.8% reduction). Motor parameters also demonstrated grade-dependent deterioration, with peroneal CMAP amplitude declining from 4.8 +/– 1.6 mV to 2.8 +/– 1.0 mV (41.7% reduction). Patients who subsequently required neurotoxicity-related dose modification had lower end-of-treatment sural SNAP amplitudes than those without treatment change (4.1 +/– 2.3 uV vs. 8.3 +/– 4.6 uV, *P* < 0.001).

### 3.4 Correlation between NCS parameters and clinical severity measures

**Figure 2:**
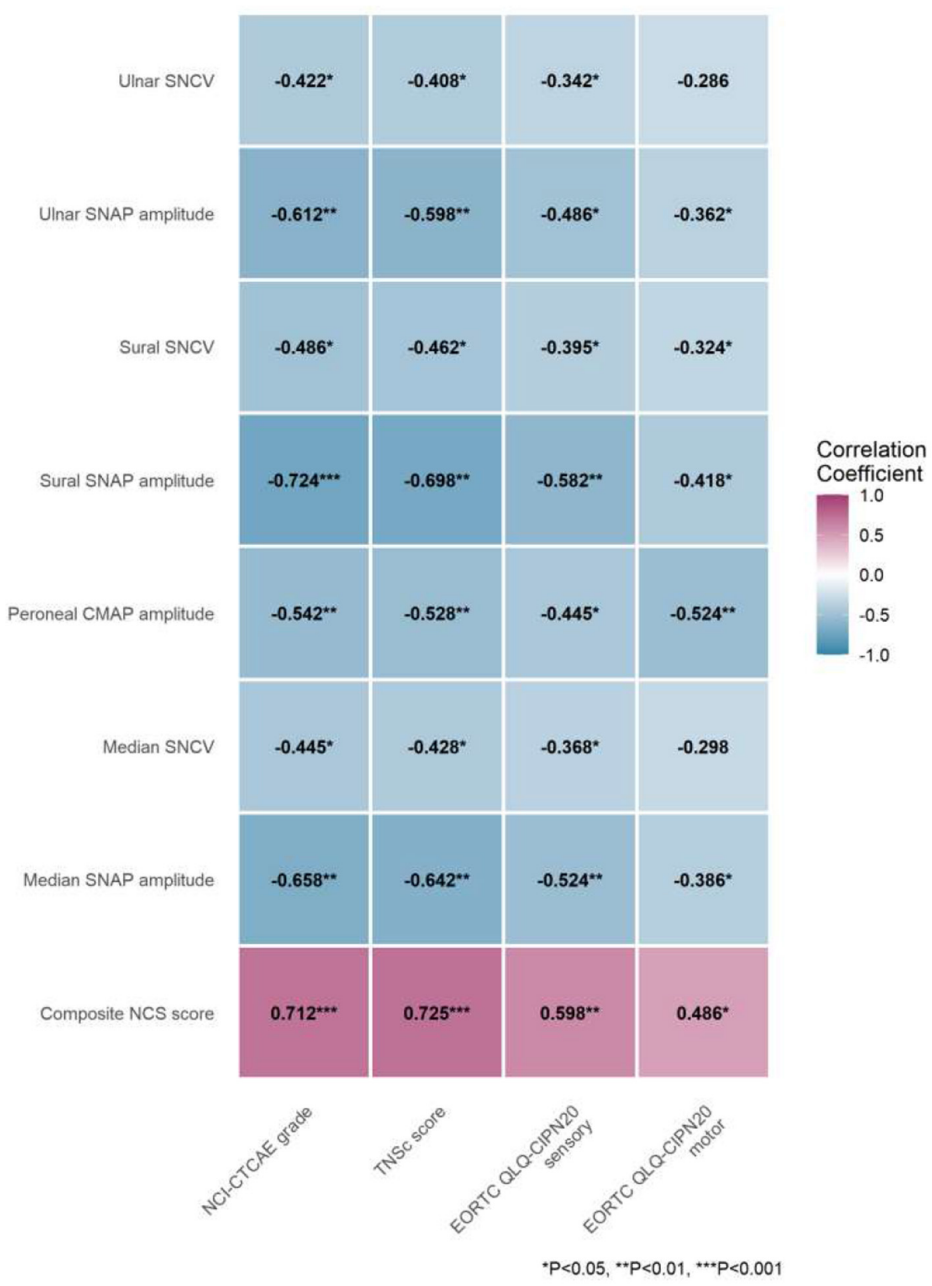
Correlation matrix between nerve conduction parameters and clinical severity measures.

Figure 2 illustrates the correlation matrix generated from paired end-of-treatment clinical and NCS assessments completed within the same visit window. Sural SNAP amplitude demonstrated the strongest negative correlation with NCI-CTCAE grade (*r* = −0.724, *P* < 0.001), followed by the composite NCS score (*r* = 0.712, *P* < 0.001), median SNAP amplitude (*r* = −0.641, *P* < 0.001), and peroneal CMAP amplitude (*r* = −0.598, *P* < 0.001).

Similar correlation patterns were observed for TNSc scores, with sural SNAP amplitude (*r* = −0.698, *P* < 0.001) and composite NCS score (*r* = 0.725, *P* < 0.001) showing the strongest associations. The EORTC QLQ-CIPN20 sensory subscale correlated moderately with sural SNAP amplitude (*r* = −0.582, *P* < 0.001), while correlations with motor subscale scores were weaker (*r* = −0.324 to −0.418).

Subgroup analyses by chemotherapy type revealed distinct correlation patterns. In patients receiving taxane-based therapy, the correlation between sural SNAP amplitude and clinical severity was strongest (*r* = −0.756, *P* < 0.001), while platinum-treated patients showed relatively stronger correlations for median and ulnar SNAP amplitudes (*r* = −0.682 and −0.664, respectively), consistent with the non-length-dependent pattern characteristic of platinum-induced sensory neuronopathy.

### 3.5 Diagnostic performance of NCS parameters for severe CIPN

**Figure 3:**
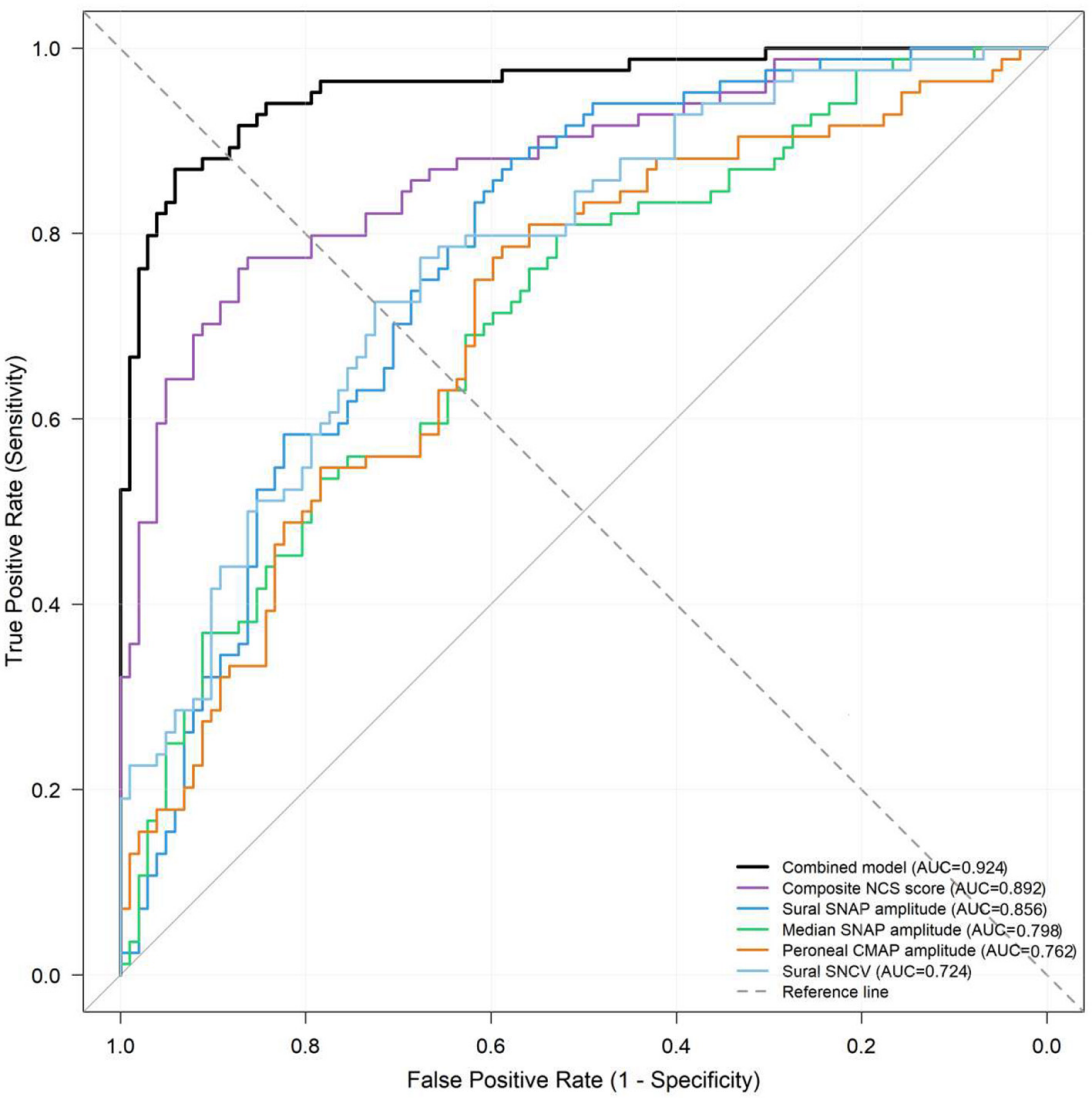
Receiver operating characteristic curves for NCS parameters in predicting severe CIPN.

ROC curve analysis was performed to evaluate the discriminative ability of end-of-treatment NCS parameters for identifying end-of-treatment Grade >= 2 CIPN (Table 3, Figure 3). Sural SNAP amplitude demonstrated excellent discrimination with an AUC of 0.856 (95% CI: 0.802–0.910). The optimal absolute cutoff value of 6.5 uV yielded sensitivity of 82.1% and specificity of 76.5%. When sural SNAP change from baseline was analyzed, a decline of >=35% at end-of-treatment yielded an AUC of 0.872 (95% CI: 0.821–0.923), with 80.4% sensitivity and 79.6% specificity, improving cross-center interpretability relative to a single absolute threshold.

**Table 3:** Diagnostic performance of NCS parameters for identifying Grade ≥2 CIPN.

Parameter	AUC (95% CI)	Cutoff	Sensitivity (%)	Specificity (%)	PPV (%)	NPV (%)
Sural SNAP amplitude	0.856 (0.802–0.910)	≤ 6.5 μV	82.1	76.5	74.3	83.8
Sural SNCV	0.724 (0.654–0.794)	≤ 45.0 m/s	64.3	72.5	65.8	71.2
Median SNAP amplitude	0.798 (0.738–0.858)	≤ 14.0 μV	72.6	74.5	70.1	76.8
Peroneal CMAP amplitude	0.762 (0.698–0.826)	≤ 3.5 mV	67.9	73.5	67.8	73.6
Composite NCS score	0.892 (0.847–0.937)	≥6	85.7	81.4	79.2	87.4
Relative sural SNAP decline from baseline	0.872 (0.821–0.923)	>=35%	80.4	79.6	77.8	81.8
Combined model^*^	0.931 (0.895–0.967)	–	88.4	84.9	82.9	89.7

^*^Combined model includes sural SNAP amplitude, median SNAP amplitude, peroneal CMAP amplitude, and composite NCS score.

AUC, area under the curve; CI, confidence interval; CIPN, chemotherapy-induced peripheral neuropathy; CMAP, compound muscle action potential; NCS, nerve conduction studies; NPV, negative predictive value; PPV, positive predictive value; SNAP, sensory nerve action potential; SNCV, sensory nerve conduction velocity.

A combined model incorporating sural SNAP amplitude, percentage sural SNAP decline from baseline, median SNAP amplitude, and peroneal CMAP amplitude achieved an AUC of 0.931 (95% CI: 0.895–0.967), significantly outperforming individual absolute parameters (*P* < 0.01 for all comparisons by DeLong's test). Because this was a retrospective single-center analysis, these thresholds should be interpreted as internally derived classification cut points rather than externally validated diagnostic biomarkers.

### 3.6 Electrophysiological phenotypes and clinical outcomes

**Figure 4:**
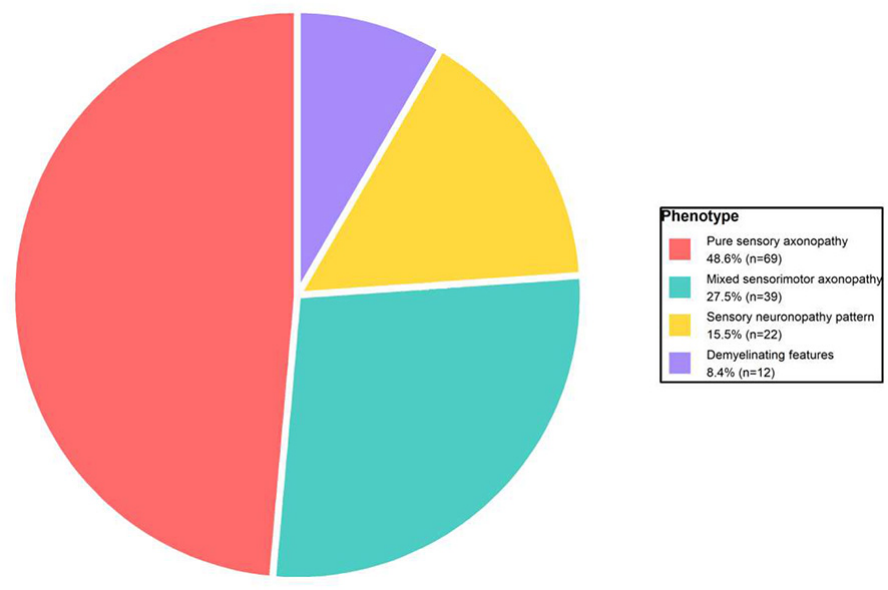
Electrophysiological phenotype distribution.

Among 142 patients who developed CIPN, four distinct electrophysiological phenotypes were identified (Figure 4): (1) pure sensory axonopathy in 69 patients (48.6%), characterized by reduced SNAP amplitudes with preserved velocities and normal motor studies; (2) mixed sensorimotor axonopathy in 39 patients (27.5%), with concurrent sensory and motor amplitude reductions; (3) sensory neuronopathy pattern in 22 patients (15.5%), showing non-length-dependent SNAP abnormalities; and (4) demyelinating features in 12 patients (8.4%), demonstrating velocity slowing disproportionate to amplitude reduction.

Phenotype distribution differed significantly by chemotherapy type (*P* < 0.001). Taxane-treated patients predominantly exhibited pure sensory axonopathy (58.1%) and mixed sensorimotor axonopathy (30.6%), while platinum-treated patients more frequently showed sensory neuronopathy pattern (28.3%) and pure sensory axonopathy (43.5%). Patients receiving combination therapy had the highest proportion of mixed sensorimotor axonopathy (41.2%).

Clinical severity varied significantly across phenotypes (*P* < 0.001). Grade 3 CIPN was most prevalent in patients with mixed sensorimotor axonopathy (35.9%) and sensory neuronopathy pattern (31.8%), compared to pure sensory axonopathy (13.0%) and demyelinating features (16.7%). The mixed sensorimotor phenotype was associated with the highest mean TNSc score (14.2 ± 5.6) and the most substantial functional impairment as measured by EORTC QLQ-CIPN20 (58.4 ± 18.2).

### 3.7 Factors associated with severe CIPN

Multivariate logistic regression analysis identified independent factors associated with Grade >=2 CIPN (Table 4). After adjustment for demographic and clinical covariates, lower baseline sural SNAP amplitude (OR = 0.82 per uV increase, 95% CI: 0.74–0.91, *P* < 0.001), older age (OR = 1.04 per year, 95% CI: 1.01–1.08, *P* = 0.012), higher BMI (OR = 1.12 per kg/m^2^, 95% CI: 1.03–1.22, *P* = 0.008), and taxane-platinum combination therapy (OR = 2.86, 95% CI: 1.42–5.76, *P* = 0.003) were independently associated with severe CIPN. Controlled diabetes mellitus without baseline diabetic neuropathy was not independently associated with severe CIPN (OR = 1.56, 95% CI: 0.68–3.58, *P* = 0.294).

**Table 4:** Multivariate logistic regression analysis for predictors of Grade ≥2 CIPN.

Variable	Adjusted OR	95% CI	*P*-value
Age (per year increase)	1.04	1.01–1.08	0.012
Female sex	1.18	0.62–2.24	0.612
BMI (per kg/m^2^ increase)	1.12	1.03–1.22	0.008
Controlled diabetes mellitus	1.56	0.68–3.58	0.294
Baseline sural SNAP (per μV increase)	0.82	0.74–0.91	< 0.001
Baseline sural SNCV (per m/s increase)	0.94	0.87–1.02	0.124
Chemotherapy regimen (ref: taxane-only)			
Platinum-only	1.12	0.54–2.32	0.762
Taxane + Platinum	2.86	1.42–5.76	0.003
Cumulative dose (per 100 mg/m^2^ equivalent)	1.24	1.08–1.42	0.002

BMI, body mass index; CI, confidence interval; CIPN, chemotherapy-induced peripheral neuropathy; OR, odds ratio; SNAP, sensory nerve action potential; SNCV, sensory nerve conduction velocity.

The model demonstrated good discrimination (C-statistic = 0.842) and calibration (Hosmer–Lemeshow *P* = 0.624). Patients with baseline sural SNAP amplitude in the lowest quartile (< 9.2 uV) had a 3.4-fold higher odds of severe CIPN compared with those in the highest quartile (>15.6 uV), supporting an association between lower pre-treatment large-fiber reserve and subsequent clinical severity.

### 3.8 Longitudinal changes in NCS parameters and treatment tolerance

**Figure 5:**
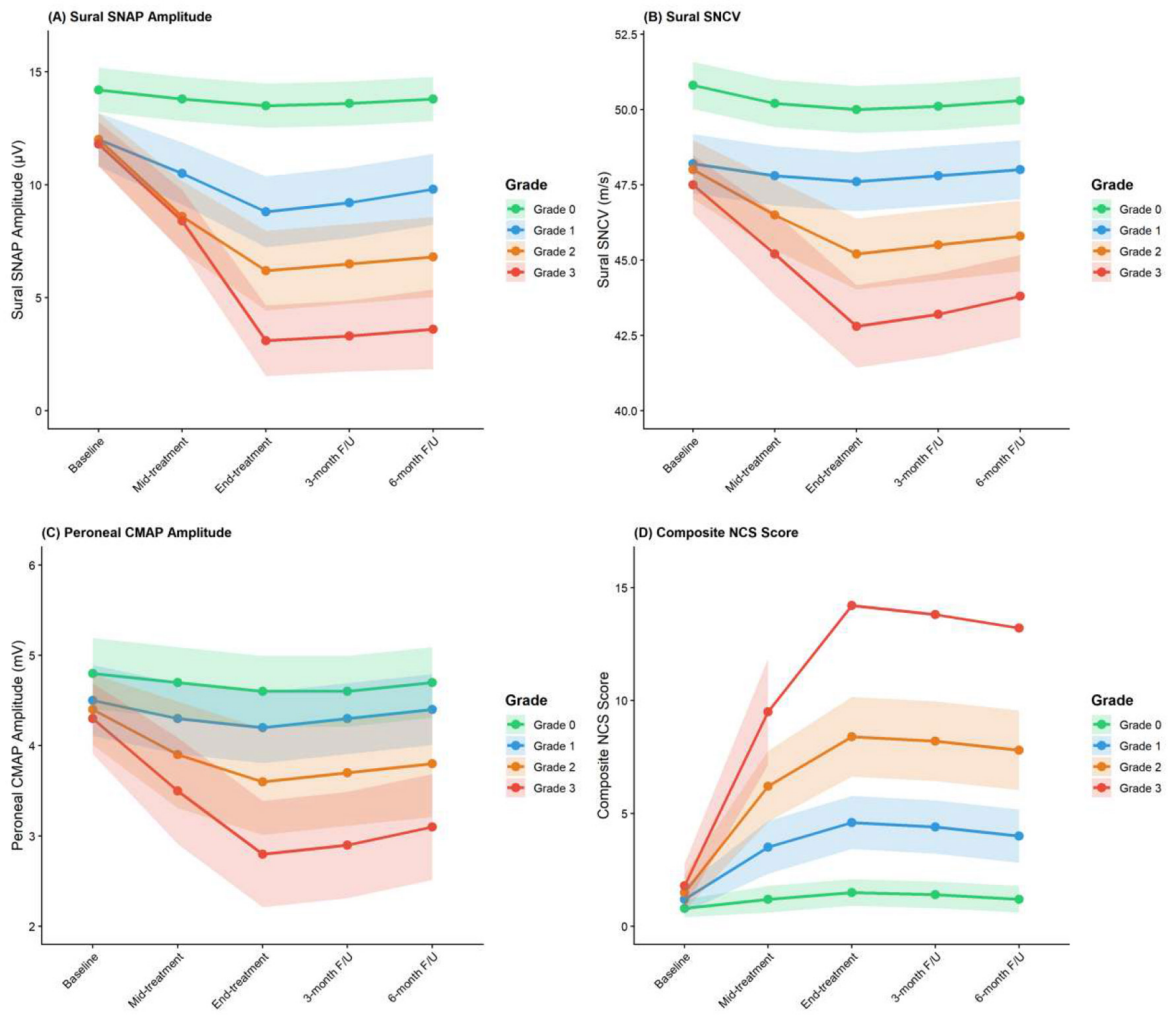
Longitudinal trajectories of nerve conduction parameters by CIPN severity group. **(A)** Sural SNAP amplitude. **(B)** Sural SNCV. **(C)** Peroneal CMAP amplitude. **(D)** Composite NCS score.

Figure 5 illustrates the trajectory of NCS parameter changes throughout the treatment course. Sural SNAP amplitude showed the earliest and most pronounced decline, with a mean reduction of 28.4% from baseline to mid-treatment and 48.6% by end-of-treatment in patients who developed Grade >=2 CIPN. In contrast, patients with Grade 0–1 CIPN showed smaller declines of 11.2% and 22.8% at the corresponding time points.

Early NCS changes from baseline to mid-treatment were also associated with subsequent treatment tolerance. A sural SNAP amplitude reduction of >=30% at mid-treatment predicted end-of-treatment Grade >=2 CIPN with sensitivity of 78.6% and specificity of 82.4% (AUC = 0.846), and it predicted neurotoxicity-related dose modification with sensitivity of 76.3% and specificity of 79.1% (AUC = 0.818). Patients who underwent dose modification had a larger early relative decline in sural SNAP amplitude than those without treatment change (34.8% +/– 13.2% vs. 17.6% +/– 11.4%, *P* < 0.001), together with higher contemporaneous TNSc scores (8.6 +/– 2.7 vs. 5.1 +/– 2.4, *P* < 0.001).

At 6-month follow-up, partial recovery was observed in most parameters, but the magnitude of recovery from end-of-treatment to 6 months was modest. Mean sural SNAP amplitude increased by 1.8 +/– 1.2 uV in Grade 1 patients, 1.1 +/– 0.9 uV in Grade 2 patients, and 0.3 +/– 0.6 uV in Grade 3 patients, corresponding to relative improvements of 12.4%, 9.0%, and 5.8%, respectively (*P* for trend = 0.021). The sensory neuronopathy phenotype showed the poorest recovery, with mean 6-month sural SNAP amplitude remaining at 48.6% of baseline, consistent with the limited regenerative capacity of dorsal root ganglion injury.

## 4 Discussion

This retrospective cohort study shows that longitudinal large-fiber NCS abnormalities were strongly associated with clinical severity grading in a clinically referred CIPN cohort, with sural SNAP amplitude demonstrating the closest relationship to symptom burden. The data also support clinically relevant heterogeneity in electrophysiological phenotype and recovery pattern, although the study primarily refines and longitudinally contextualizes established observations rather than introducing a fully new disease classification.

The high incidence of CIPN (76.3%) in our cohort is compatible with prior reports documenting neuropathy rates of 30%−70%, while also reflecting referral enrichment because only patients undergoing NCS were included (16). This referral pattern likely selected patients with more symptomatic, persistent, or clinically ambiguous neuropathy, and our findings should therefore be generalized most cautiously to routine oncology populations without electrophysiological referral (16, 17).

Our observation that sural SNAP amplitude demonstrated the strongest correlation with clinical severity extends earlier work identifying distal sensory amplitudes as robust objective correlates of CIPN (17, 18). The accompanying percentage-change analysis is clinically useful because it reduces dependence on center-specific normative values and better contextualizes deterioration for patients who begin treatment with either low-normal or high-normal baseline responses.

The discriminative performance of serial NCS for classifying severe CIPN was notable, but the present data should be interpreted as associative rather than practice-changing. Our cohort did not undergo prospective NCS-guided intervention, and the internally derived cutoffs should not be considered validated standalone biomarkers or mandatory thresholds for chemotherapy modification without external replication (12, 19).

The four electrophysiological patterns described here are best viewed as pragmatic descriptors of large-fiber involvement rather than definitive new CIPN subtypes. Even so, the observed distribution across chemotherapy regimens and the differences in recovery support the clinical relevance of documenting whether a patient shows predominantly length-dependent sensory axonopathy, mixed sensorimotor involvement, or a neuronopathic pattern.

The association between electrophysiological pattern and outcome was most evident for mixed sensorimotor axonopathy and sensory neuronopathy, which showed greater severity and less recovery. This finding is mechanistically plausible and may help frame prognosis during follow-up, but it still requires confirmation in prospective cohorts before being used as a formal prognostic schema (20–22).

Lower baseline sural SNAP amplitude and larger early decline from baseline were associated with severe CIPN and with neurotoxicity-related dose modification. These results suggest that serial NCS may help identify patients with limited large-fiber reserve or rapidly evolving neurotoxicity, but they do not demonstrate that acting on NCS findings would improve oncologic or neurologic outcomes.

The identification of older age, higher BMI, and combination chemotherapy as factors associated with severe CIPN is consistent with prior literature (23, 24). These variables likely capture differences in cumulative neurotoxic exposure, physiologic reserve, and metabolic vulnerability rather than isolated causal pathways.

Several limitations warrant consideration. First, the retrospective single-center design introduces selection bias because NCS referral was symptom-driven rather than universal. Second, conventional NCS captures large-fiber dysfunction and cannot adequately detect early small-fiber injury, which may explain discordance between symptoms and electrophysiology in some patients (12, 25). Third, NCS findings were not used prospectively to guide treatment, so the study cannot establish the benefit of NCS-directed dose modification. Fourth, missing data at later follow-up visits may have influenced recovery estimates. Finally, the 6-month observation window does not capture the full long-term course of CIPN recovery.

Future work should focus on prospective multicenter validation of relative NCS change thresholds, integration of large-fiber electrophysiology with small-fiber measures and patient-reported outcomes, and formal testing of whether serial neurophysiological monitoring can safely improve chemotherapy management.

## 5 Conclusions

This study demonstrates that longitudinal NCS abnormality patterns are closely associated with clinical severity grading in a referred CIPN cohort. Sural SNAP amplitude, particularly its percentage decline from baseline, showed the strongest association with severe neuropathy and with neurotoxicity-related dose modification. Electrophysiological phenotyping also captured clinically relevant heterogeneity in severity and recovery. These findings support serial NCS as an objective adjunct for CIPN assessment, while prospective external validation remains necessary before specific thresholds are used for treatment decisions.

## Data Availability

The original contributions presented in the study are included in the article/supplementary material, further inquiries can be directed to the corresponding author.
